# High genetic similarity between *Clostridioides difficile* isolates from a woman with community-acquired infection and her dog

**DOI:** 10.3389/fpubh.2025.1755562

**Published:** 2026-01-09

**Authors:** Júlia Meireles, Suzana Leite, Amanda Macedo Trindade de Castro Brandt, Geraldo Alves Neto, Rodrigo Otávio Silveira Silva, Eliane de Oliveira Ferreira

**Affiliations:** 1 Laboratory of Anaerobes Biology, Department of Medical Microbiology, Instituto de Microbiologia Paulo de Góes, Universidade Federal do Rio de Janeiro, Rio de Janeiro, Brazil; 2 Santa Úrsula University, Rio de Janeiro, Brazil; 3 Autonomous Veterinary, Rio de Janeiro, Brazil; 4 Veterinary School, Universidade Federal de Minas Gerais, Belo Horizonte, Brazil

**Keywords:** *Clostridioides difficile*, community-acquired infection, companion animals, genomic similarity, potential transmission

## Abstract

*Clostridioides difficile* infection is a leading cause of diarrhea associated with healthcare settings, but its incidence in the community has increased substantially in recent years, raising questions about possible non-hospital reservoirs. We report a case of community-acquired *C. difficile* infection in a woman and the concurrent isolation of a toxigenic strain from her dog, which also presented diarrhea. Both isolates showed the same ribotype and differed by only four single-nucleotide polymorphisms in whole-genome sequencing analysis, indicating a high degree of genetic similarity. These findings suggest the possibility of shared exposure or transmission between the patient and her dog and highlight the need to further investigate companion animals as potential sources of community-acquired *C. difficile*.

## 1 Introduction

*Clostridioides difficile* is a Gram-positive, strictly anaerobic, spore-forming bacillus that represents the leading cause of antibiotic-associated diarrhea in humans worldwide (1). Although traditionally linked to healthcare settings, an increasing number of community-acquired *C. difficile* infections (CA-CDI) have been reported. This epidemiological shift has prompted the scientific community to investigate the underlying factors contributing to the rising incidence of CDI, highlighting potential sources of *C. difficile* spore dissemination beyond hospital environments. Among the proposed sources are contaminated food products and animals, which may act as reservoirs for toxigenic *C. difficile* strains.

Most studies to date have focused on food-producing animals, whereas relatively few have examined the role of companion animals (2–4). Increasing evidence supports the circulation of genetically related *C. difficile* strains across humans, animals and environmental sources. A recent One Health genomic investigation conducted in Flagstaff, USA, analyzed more than 500 isolates and identified substantial genomic overlap among clinical, canine and environmental reservoirs, including several ST42 lineages. Using a ≤ 2-SNP threshold, the study detected multiple putative transmission events, highlighting the importance of evaluating companion animals as potential contributors to community-associated CDI dynamics (5). Notably, *C. difficile* strains identical to those implicated in human CDI cases have been isolated from dogs, animals that share close contact with humans and frequently interact within shared household environments (6–8). More recently, Garza et al. (9) reported a recurrent case of CA-CDI suspected to have originated from a household cat, further underscoring the potential role of pets as sources of infection, particularly among high-risk individuals.

Given this context, investigating the epidemiology of *C. difficile* in companion animals may yield valuable insights into the transmission dynamics of CA-CDI and support the development of more effective preventive strategies. In this study, we describe a case of CA-CDI and the concurrent isolation of a *C. difficile* strain with high genetic similarity from the patient’s dog, which had also presented episodes of diarrhea.

## 2 Case report

In August 2013, a 27-year-old woman was diagnosed with idiopathic ulcerative colitis. Two years later, following an episode of diarrhea, a stool sample was tested for *C. difficile* A/B toxins, which yielded a negative result. In January 2021, the patient experienced a worsening of intestinal inflammation, for which antibiotic therapy with ciprofloxacin and metronidazole was initiated. Despite treatment, her condition continued to deteriorate, leading to hospitalization, where she received corticosteroids and amoxicillin-clavulanate, and was subsequently discharged.

Four months later (May 2021), the patient presented to the hospital with severe abdominal pain, abdominal distension, and bloody diarrhea. Colonoscopy confirmed pseudomembranous colitis, and a stool sample tested positive for *C. difficile* A/B toxins. The patient reported owning three dogs, one of which also exhibited diarrhea. Stool samples were collected from all three dogs. Recognizing that natural defecation results in unavoidable initial exposure to the environment, all samples (human and canine) were collected immediately after deposition, transferred into sterile containers, to minimize additional environmental contamination, and stored at −20 °C until processing. All samples were then subjected to *C. difficile* isolation and PCR for the detection of toxin A (*tcdA*), toxin B (*tcdB*), and binary toxin (*cdtB*) genes (10). Toxigenic isolates (*tcdA*^+^ *tcdB*^+^ *cdtB*^−^) were obtained from the patient and from one of the dogs, which presented diarrhea. The patient was subsequently treated with vancomycin and discharged after approximately 5 days, with no recurrences reported to date.

## 3 Microbiological and genomics findings

To further characterize the *C. difficile* isolates, both strains were subjected to PCR ribotyping (11) and antimicrobial susceptibility testing using the disk diffusion method against erythromycin (15 μg), metronidazole (5 μg), rifampicin (5 μg), vancomycin (5 μg), and moxifloxacin (5 μg) (12–14). Both isolates were identified as ribotype 106 (RT106) and were susceptible to all antibiotics tested.

The isolation of strains sharing the same ribotype and antimicrobial susceptibility profile from the patient and her dog raised the hypothesis of clonality. To investigate this possibility in greater detail, genomic DNA from both strains was extracted using the Wizard Genomic DNA Purification Kit (Promega, USA). Whole-genome sequencing was then performed on the Illumina MiSeq platform (mid-output 2 × 150 bp cycles), and the raw reads were analyzed with FastQC (Babraham Bioinformatics, Cambridge, UK), retaining only paired reads with a Phred quality score ≥30 and a minimum length of 50 nucleotides. Genome assembly was carried out using SPAdes 3.5.0 in careful mode (15), followed by gap filling and polishing with Pilon (16). Sequence types were determined using PubMLST (17).

Both isolates were classified as ST42, the sequence type typically associated with RT106. No antimicrobial resistance determinants were detected, corroborating the results obtained by the disk diffusion method. RT106/ST42 is among the most commonly isolated strains from CDI patients in Brazil (18–21) and has also been reported worldwide (22–24). In addition to its relevance in humans, RT106/ST42 appears to be common in dogs as well (24–29).

To further assess the similarity between these two isolates, a single nucleotide polymorphism (SNP) analysis was performed using CSIPhylogeny (29), with a minimum Z-score of 1.96 and a minimum depth at each SNP position of 10 × and using CD630 (30) as reference. For comparison, *C. difficile* ST42 from dogs and humans in Brazil were included. Then, isolates (also ST42) from other countries with a known high flow of people to and from Brazil were also added. These strains were obtained from the Bacterial and Viral Bioinformatics Resource Center (BV-BRC; https://www.bv-brc.org/) and from previous studies (30–38) (Supplementary Table S1). Reference strains from clade 2 (2,007,855, R20291, BI1 and CD196), clade 3 (M68), clade 4 (CF5) and clade 5 (M120). The phylogenetic tree was visualized using the iTOL online tool with midpoint rooting (39).

**Figure 1:**
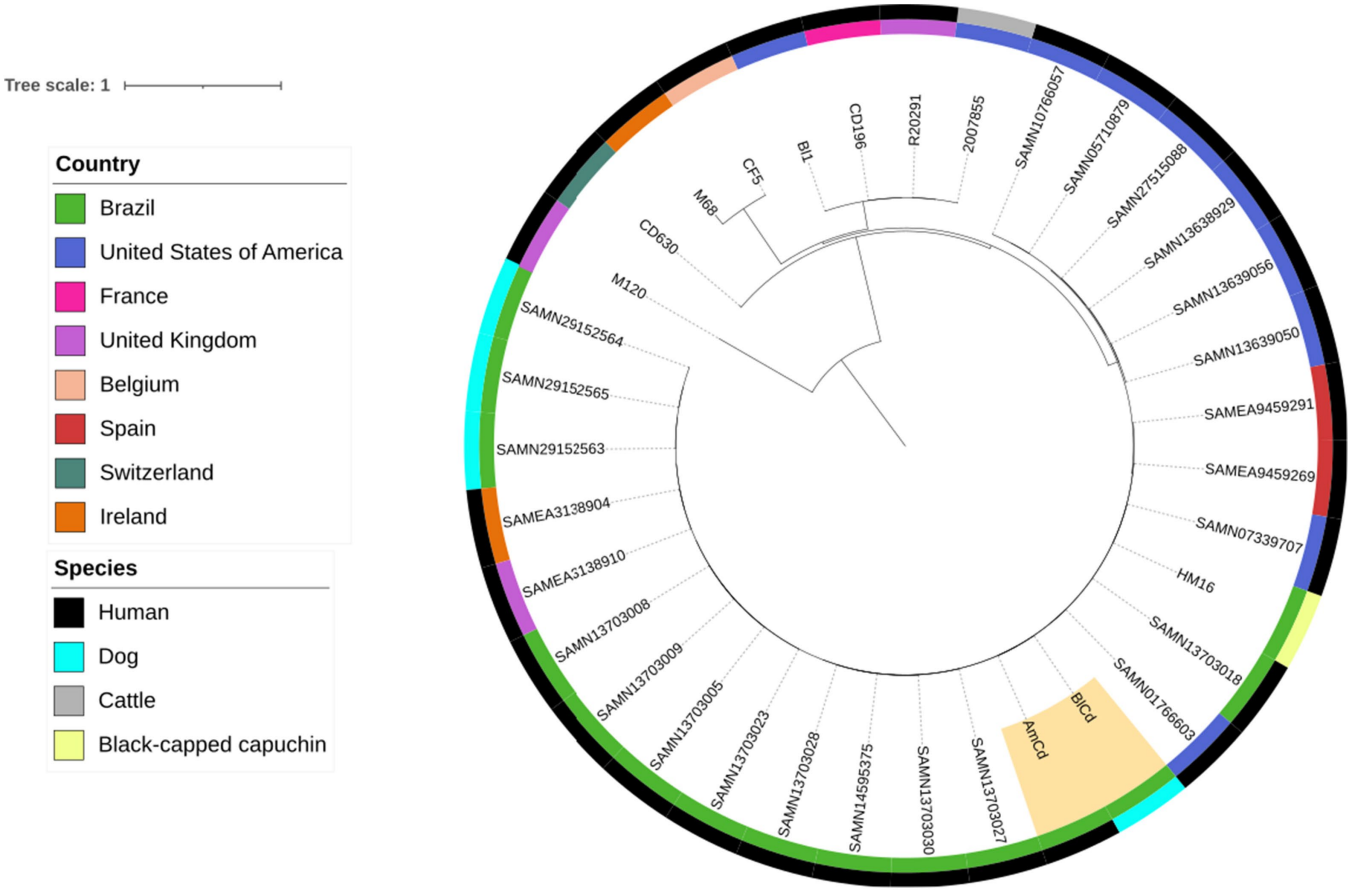
Phylogenetic tree of 27 *C. difficile* ST42 isolates from canine and humans. The strains from the present study (BlCd and AmCd are highlighted in beige). Reference strains from clade 1 (630), clade 2 (2007855, R20291, BI1, and CD196), clade 3 (M68), clade 4 (CF5), and clade 5 (M120) were included for comparison purpose.

SNP analysis encompassed up to 98% of the included genomes and demonstrated that the two isolates from this study differed by only four SNPs (Supplementary Table S2). Such high genetic similarity strongly supports the hypothesis of potential clonality, as reported in previous studies (40, 41). These findings indicate a possible transmission of *C. difficile* between the patient and her dog (see Figure 1).

## 4 Discussion

The incidence of CA-CDI has nearly doubled over the past decade, with some regions reporting that up to half of all CDI cases in certain countries originate in the community rather than in healthcare settings (42). Furthermore, CA-CDI increasingly affects younger and otherwise healthy individuals, often in the absence of traditional risk factors such as recent hospitalization or antibiotic use (43). This epidemiological shift highlights the importance of investigating non-traditional sources of infection in the transmission dynamics of the disease.

The present study further supports the potential role of household animals as reservoirs by demonstrating a high degree of genomic similarity between *C. difficile* strains isolated from a woman with CA-CDI and her dog, differing by only four SNPs, well within the range considered indicative of potential clonality (44). Notably, the only dog that tested positive for *C. difficile* was also the only one that shared the bed with the patient. Recently, Garza et al. (9) provided evidence suggesting that a cat may have acted as a reservoir in a case of recurrent CDI in a woman, who achieved clinical resolution only after the animal was also treated. Indeed, previous studies from different countries have raised the hypothesis that dogs may serve as reservoirs of toxigenic *C. difficile* for humans, based on the high similarity of isolates from these animals and strains causing CDI in humans (6, 7, 10, 44, 45).

However, this study has some limitations. First, it is impossible to determine whether the diarrhea observed in the dog was caused by *C. difficile*, as no additional diagnostic testing was performed. Research indicates that while dogs carrying *C. difficile* often show gut microbial imbalance and reduced *C. hiranonis* levels, which can alter bile acid metabolism, its presence appears to play a minor role in canine clinical gastrointestinal diseases (46). Consequently, although studies on *C. difficile* in pets are increasing, its true impact on canine health remains uncertain.

Furthermore, the direction of transmission cannot be established, and transmission itself remains unconfirmed, since our approach demonstrates the possibility rather than providing definitive evidence. Another important consideration is that colonization of the dog by the isolated strain cannot be confirmed; therefore, it is possible that the isolate represents only a transient presence of *C. difficile* in the dog’s feces. Nevertheless, this work contributes to the understanding of the complex epidemiology of CA-CDI and suggests that companion animals may serve as reservoirs of toxigenic *C. difficile* for humans, which could have particular relevance for individuals with predisposing risk factors.

## Data Availability

The original contributions presented in the study are included in the article/Supplementary material, further inquiries can be directed to the corresponding author.
