# Supplementary material for: High genetic similarity between Clostridioides difficile isolates from a woman with community-acquired infection and her dog
Source: Front Public Health. 2026 Jan 9;13:1755562. doi: 10.3389/fpubh.2025.1755562 (PMC12827726; doi:10.3389/fpubh.2025.1755562)
Supplement: Supplementary file 1 [file Table_1.docx]

**Supplementary table 1:** *C. difficile* strains used for the SNP analysis

| Strain - acession number | Host | Country | ST | Reference |
| --- | --- | --- | --- | --- |
| AmCd (SAMN53882509) | Human | Brazil | 42 | Current study |
| BlCd (SAMN53882510) | Dog | Brazil | 42 | Current study |
| HM16 (SAMN53421074) | *Sapajus apella* | Brazil | 42 | Current study |
| SAMN13703028 | Human | Brazil | 42 | Girão et al. 2021 |
| SAMN13703027 | Human | Brazil | 42 | Girão et al. 2021 |
| SAMN13703009 | Human | Brazil | 42 | Girão et al. 2021 |
| SAMN13703008 | Human | Brazil | 42 | Girão et al. 2021 |
| SAMN13703018 | Human | Brazil | 42 | Girão et al. 2021 |
| SAMN13703023 | Human | Brazil | 42 | Girão et al. 2021 |
| SAMN13703030 | Human | Brazil | 42 | Girão et al. 2021 |
| SAMN14595375 | Human | Brazil | 42 | Girão et al. 2021 |
| SAMN13703005 | Human | Brazil | 42 | Girão et al. 2021 |
| SAMN29152563 | Dog | Brazil | 42 | Rainha et al. 2019 |
| SAMN29152564 | Dog | Brazil | 42 | Rainha et al. 2019 |
| SAMN29152565 | Dog | Brazil | 42 | Rainha et al. 2019 |
| SAMEA3138910 | Human | UK | 42 | Kurka et al. 2014 |
| SAMEA3138904 | Human | Ireland | 42 | Kurka et al. 2014 |
| SAMEA9459291 | Human | Spain | 42 | Suárz-Bode et al. 2023 |
| SAMEA9459269 | Human | Spain | 42 | Suárez-Bode et al. 2023 |
| SAMN07339707 | Human | USA | 42 | Ozer et al. 2017 |
| SAMN01766603 | Human | USA | 42 | Not applicable |
| SAMN05710879 | Human | USA | 42 | Williamson et al. 2023 |
| SAMN10766057 | Human | USA | 42 | Williamson et al. 2023 |
| SAMN13638929 | Human | USA | 42 | Williamson et al. 2023 |
| SAMN13639050 | Human | USA | 42 | Williamson et al. 2023 |
| SAMN13639056 | Human | USA | 42 | Williamson et al. 2023 |
| SAMN27515088 | Human | USA | 42 | Williamson et al. 2023 |
| 2007855 (SAMEA3138369) | Cattle | USA | 1 | Not applicable |
| BI1 (SAMEA3138395) | Human | USA | 1 | He et al. 2010 |
| CD196 (SAMEA3138290) | Human | France | 1 | Stabler et al. 2009 |
| CD630 (SAMEA1705932) | Human | Switzerland | 54 | Sebaihia et al. 2006 |
| CF5 (SAMEA3138367) | Human | Belgium | 86 | He et al. 2010 |
| M68 (SAMEA3138366) | Human | Ireland | 37 | He et al. 2010 |
| M120 (SAMEA3138368) | Human | UK | 11 | He et al. 2010 |
| R20291 (SAMEA3138243) | Human | UK | 1 | Stabler et al. 2009 |
