# Supplementary material for: High genetic similarity between Clostridioides difficile isolates from a woman with community-acquired infection and her dog
Source: Front Public Health. 2026 Jan 9;13:1755562. doi: 10.3389/fpubh.2025.1755562 (PMC12827726; doi:10.3389/fpubh.2025.1755562)
Supplement: Supplementary file 2 [file Table_2.pdf]

	2007855	AmCd	Bl1	BlCd
2007855	0	12874	206	12874
AmCd	12874	0	12722	4
Bl1	206	12722	0	12722
BlCd	12874	4	12722	0
CD196	190	12730	24	12730
CF5	23909	21723	23791	21721
HM16	12870	68	12700	66
M120	42402	39944	42337	39942
M68	26265	24885	26147	24883
R20291	112	12818	132	12818
SAMEA3138904	12891	132	12741	132
SAMEA3138910	12888	106	12738	106
SAMEA9459269	12848	62	12698	60
SAMEA9459291	12868	78	12716	78
SAMN01766603	12865	27	12713	27
SAMN05710879	12906	789	12754	787
SAMN07339707	12863	49	12693	47
SAMN10766057	12909	716	12755	716
SAMN13638929	12889	894	12719	892
SAMN13639050	12882	865	12730	863
SAMN13639056	12883	866	12731	864
SAMN13703005	12936	144	12784	144
SAMN13703008	12910	149	12740	149
SAMN13703009	12905	96	12753	96
SAMN13703018	12872	38	12720	38
SAMN13703023	12907	77	12753	75
SAMN13703027	12881	38	12729	38
SAMN13703028	12880	38	12728	36
SAMN13703030	12879	35	12727	33
SAMN14595375	12881	53	12731	51
SAMN27515088	12872	847	12702	845
SAMN29152563	12916	114	12764	114
SAMN29152564	12896	82	12744	80
SAMN29152565	12908	96	12756	94
CD630	12331	6212	12141	6212

min: 4 max: 45209

CD196	CF5	HM16	M120	M68
190	23909	12870	42402	26265
12730	21723	68	39944	24885
24	23791	12700	42337	26147
12730	21721	66	39942	24883
0	23795	12708	42340	26151
23795	0	21699	44328	4243
12708	21699	0	39930	24861
42340	44328	39930	0	45209
26151	4243	24861	45209	0
124	23837	12796	42380	26193
12749	21739	144	39961	24899
12746	21736	118	39960	24896
12708	21701	92	39920	24861
12724	21707	112	39936	24869
12721	21714	63	39933	24874
12762	21721	817	39973	24875
12701	21694	41	39927	24856
12765	21736	748	39998	24889
12727	21679	884	39902	24818
12738	21690	891	39903	24829
12739	21691	892	39904	24830
12792	21777	182	40008	24940
12748	21734	129	39974	24895
12761	21743	130	39975	24904
12728	21721	66	39940	24881
12763	21742	113	39971	24901
12737	21732	76	39945	24891
12736	21727	72	39943	24886
12735	21728	73	39944	24887
12739	21732	89	39950	24893
12710	21667	835	39925	24822
12774	21755	134	39989	24914
12754	21733	100	39969	24894
12766	21743	114	39974	24904
12151	20411	6198	37230	23354

R20291	SAMEA3138904	SAMEA3138910	SAMEA9459269	SAMEA9459291
112	12891	12888	12848	12868
12818	132	106	62	78
132	12741	12738	12698	12716
12818	132	106	60	78
124	12749	12746	12708	12724
23837	21739	21736	21701	21707
12796	144	118	92	112
42380	39961	39960	39920	39936
26193	24899	24896	24861	24869
0	12834	12831	12792	12812
12834	0	62	153	132
12831	62	0	127	106
12792	153	127	0	62
12812	132	106	62	0
12809	121	93	57	73
12848	812	814	813	797
12789	147	121	75	95
12851	765	739	740	726
12813	943	915	906	890
12826	913	885	877	859
12827	914	886	878	860
12878	163	165	172	146
12834	152	154	175	147
12847	119	121	124	94
12816	130	104	64	82
12849	130	104	103	79
12823	139	113	70	86
12822	137	111	66	84
12821	136	110	65	85
12823	124	126	81	101
12796	894	868	813	807
12860	145	123	144	116
12840	119	93	110	84
12852	133	107	124	98
12257	6263	6250	6180	6203

SAMN01766603	SAMN05710879	SAMN07339707	SAMN10766057	SAMN13638929
12865	12906	12863	12909	12889
27	789	49	716	894
12713	12754	12693	12755	12719
27	787	47	716	892
12721	12762	12701	12765	12727
21714	21721	21694	21736	21679
63	817	41	748	884
39933	39973	39927	39998	39902
24874	24875	24856	24889	24818
12809	12848	12789	12851	12813
121	812	147	765	943
93	814	121	739	915
57	813	75	740	906
73	797	95	726	890
0	786	46	711	889
786	0	794	129	323
46	794	0	731	867
711	129	731	0	238
889	323	867	238	0
858	322	876	229	37
859	323	877	230	38
139	835	165	788	966
144	834	124	795	933
89	779	107	740	916
31	789	51	718	894
74	794	94	723	899
35	795	57	724	900
33	791	55	722	896
32	792	54	721	897
50	780	70	739	913
840	252	820	183	261
109	831	133	748	936
79	797	99	716	902
93	811	113	730	916
6201	6201	6189	6236	6018

SAMN13639050	SAMN13639056	SAMN13703005	SAMN13703008	SAMN13703009
12882	12883	12936	12910	12905
865	866	144	149	96
12730	12731	12784	12740	12753
863	864	144	149	96
12738	12739	12792	12748	12761
21690	21691	21777	21734	21743
891	892	182	129	130
39903	39904	40008	39974	39975
24829	24830	24940	24895	24904
12826	12827	12878	12834	12847
913	914	163	152	119
885	886	165	154	121
877	878	172	175	124
859	860	146	147	94
858	859	139	144	89
322	323	835	834	779
876	877	165	124	107
229	230	788	795	740
37	38	966	933	916
0	7	939	942	887
7	0	940	943	888
939	940	0	167	110
942	943	167	0	101
887	888	110	101	0
863	864	144	153	92
872	873	115	138	83
873	874	126	145	90
869	870	122	143	88
870	871	121	142	89
886	887	107	132	73
270	271	919	884	867
909	910	162	153	116
875	876	128	125	80
889	890	140	137	94
6029	6030	6294	6263	6264

SAMN13703018	SAMN13703023	SAMN13703027	SAMN13703028	SAMN13703030
12872	12907	12881	12880	12879
38	77	38	38	35
12720	12753	12729	12728	12727
38	75	38	36	33
12728	12763	12737	12736	12735
21721	21742	21732	21727	21728
66	113	76	72	73
39940	39971	39945	39943	39944
24881	24901	24891	24886	24887
12816	12849	12823	12822	12821
130	130	139	137	136
104	104	113	111	110
64	103	70	66	65
82	79	86	84	85
31	74	35	33	32
789	794	795	791	792
51	94	57	55	54
718	723	724	722	721
894	899	900	896	897
863	872	873	869	870
864	873	874	870	871
144	115	126	122	121
153	138	145	143	142
92	83	90	88	89
0	83	44	42	43
83	0	55	51	50
44	55	0	16	15
42	51	16	0	11
43	50	15	11	0
55	66	31	27	26
845	850	853	849	850
118	97	106	106	105
84	65	76	74	73
98	79	90	88	87
6210	6249	6218	6218	6217

SAMN14595375	SAMN27515088	SAMN29152563	SAMN29152564	SAMN29152565
12881	12872	12916	12896	12908
53	847	114	82	96
12731	12702	12764	12744	12756
51	845	114	80	94
12739	12710	12774	12754	12766
21732	21667	21755	21733	21743
89	835	134	100	114
39950	39925	39989	39969	39974
24893	24822	24914	24894	24904
12823	12796	12860	12840	12852
124	894	145	119	133
126	868	123	93	107
81	813	144	110	124
101	807	116	84	98
50	840	109	79	93
780	252	831	797	811
70	820	133	99	113
739	183	748	716	730
913	261	936	902	916
886	270	909	875	889
887	271	910	876	890
107	919	162	128	140
132	884	153	125	137
73	867	116	80	94
55	845	118	84	98
66	850	97	65	79
31	853	106	76	90
27	849	106	74	88
26	850	105	73	87
0	866	121	85	99
866	0	889	855	869
121	889	0	46	58
85	855	46	0	20
99	869	58	20	0
6233	6097	6266	6248	6254

CD630
12331
6212
12141
6212
12151
20411
6198
37230
23354
12257
6263
6250
6180
6203
6201
6201
6189
6236
6018
6029
6030
6294
6263
6264
6210
6249
6218
6218
6217
6233
6097
6266
6248
6254
0
